# Challenging the Cinderella Hypothesis: A New Model for the Role of the Motor Unit Recruitment Pattern in the Pathogenesis of Myofascial Pain Syndrome in Postural Muscles

**DOI:** 10.5041/RMMJ.10336

**Published:** 2018-07-30

**Authors:** Amir Minerbi, Simon Vulfsons

**Affiliations:** 1 Department of Family Medicine, Clalit Health Services, Haifa and Western Galilee District, Israel; 2 Institute for Pain Medicine, Rambam Health Care Campus, Haifa, Israel; 3 Bruce Rappaport Faculty of Medicine, Technion, Haifa, Israel

**Keywords:** Energy crisis, motor unit rotation, myofascial pain, shift model

## Abstract

**Background:**

The energy crisis hypothesis, which is a widely accepted model for the pathogenesis of myofascial pain, has been corroborated by experimental observations. However, the nature of the insult leading to the energy crisis remains elusive. A commonly cited model for this insult is the Cinderella hypothesis, suggesting that hierarchical recruitment of motor units leads to a disproportional load on small units, thus driving them towards an energy crisis. New findings cast doubt on this model, showing that in postural muscles motor units are recruited in rotation, rather than in a hierarchical order, precluding the formation of the so-called Cinderella units.

**Objective:**

To explore the influence of common myofascial predisposing factors such as muscle load and muscle strength on the relaxation time of postural muscle motor units, assuming they are recruited in rotation.

**Methods:**

A stochastic model of a postural skeletal muscle was developed which integrates the energy crisis model and motor unit rotation patterns observed in postural muscles. Postulating that adequate relaxation time is essential for the energetic replenishment of motor units, we explored the influence of different parameters on the relaxation time of individual motor units under varying conditions of muscle loads and muscle strengths.

**Results:**

The motor unit relaxation/contraction time ratio decreases with elevated muscle loads and with decreased total muscle strength.

**Conclusions:**

In a model of a postural muscle, in which motor units are recruited in rotation, common predisposing factors of myofascial pain, such as increased muscle load and decreased muscle force, lead to shortened motor unit relaxation periods.

## INTRODUCTION

Myofascial pain is arguably one of the most common chronic pain syndromes. It is estimated to affect the lives of millions worldwide, leading to considerable suffering, disability, and cost.^1^–^5^ Although it was recognized and studied decades ago, and despite significant progress achieved by recent studies, the pathophysiology of the syndrome remains elusive.

Myofascial pain typically arises in skeletal muscles and may refer pain to other areas of the body.^1^,^6^ Myofascial pain often involves postural muscles. These are typically proximal, tonic muscles, which act to maintain the body posture in the gravitational field. The risk for developing the syndrome is thought to increase with muscle load, either acute or chronic.^6^–^12^ Despite the paucity of published data on the individual prevalence of myofascial pain in different muscles, in our clinical experience postural tonic muscles, such as the deep back, gluteal, and cervical muscles, seem to be involved more often than others.

### The Energy Crisis Hypothesis

One of the most widely accepted theories for the pathophysiology of myofascial pain is the *energy crisis hypothesis*. This hypothesis, suggested by Simons and Travell, postulates a vicious cycle initiated by a primary insult to the muscle which initiates a cascade of events eventually leading to prolonged contraction.^13^

Normally, an action potential reaching the neuromuscular junction results in the release of Ca^2+^ from the endoplasmic reticulum to the myoplasm, thus leading to the activation of the actin-myosin contractile system. During the relaxation of a muscle fiber, Ca^2+^ is pumped back into the endoplasmic reticulum, thus allowing motor proteins to dissociate in an ATP-dependent process.^14^–^16^

An energy crisis is thought to occur when the muscle energy consumption exceeds its energy supply. Several precipitating factors have been suggested,^13^–^15^ but their common endpoint is prolonged actin-myosin coupling, leading to muscle fiber contraction and to increased resistance to flow in the microvascular bed of the contracted muscle. The arteriolar and capillary constriction, possibly aggravated by a local vasoconstrictor reflex, impairs blood flow to the tense muscle fibers, thus leading to decreased levels of oxygen and glucose, resulting in decreased ATP regeneration.^17^–^21^ The latter may interfere with the reuptake of Ca^2+^ into the sarcoplasmic reticulum, a process which is, at least in part, ATP-dependent, thus prolonging actin-myosin cross-bridging and inciting a vicious cycle.^13^

In short, an energy crisis is caused by a simultaneous increase in energy consumption, due to impaired relaxation, and a decreased energy replenishment, due to impaired blood flow in the microvasculature of the contracted muscle fibers.

Recent studies have experimentally corroborated the energy crisis hypothesis: exploring the milieu of trigger points using microdialysis pumps, Shah and colleagues have been able to demonstrate an acidic, hypoxic environment, rich in neuropeptides and inflammatory mediators.^22^,^23^

However, the energy crisis hypothesis leaves a significant unanswered question: *What causes an otherwise normal muscle to experience an energy crisis?*

### The Cinderella Hypothesis

Since affected patients are often young and healthy, and since no reproducible morphological anomaly can be found on pathological specimens, it is unlikely that a structural lesion (such as atherosclerosis) is responsible for the development of an energy crisis; rather, the cause may be dynamic, reversible interference with the muscle’s energetic replenishment mechanism.

For that reason, some research has focused on the recruitment of motor units within the skeletal muscle. Numerous studies have shown that motor units typically contract in a predetermined order from smallest to largest.^24^–^28^ The first units to be recruited are the smallest ones, allowing for fine tuning of the force applied in delicate tasks. Only as the load approaches maximum values are the largest units recruited. This hierarchical pattern of recruitment is often termed the “onion skin” pattern.^29^

This observation led Kadefors and colleagues to postulate the *Cinderella hypothesis*.^30^ They reasoned that since small motor units are first to be recruited and last to be relaxed during prolonged muscle contractions, these units are expected to be contracted for long periods of time. Prolonged contraction would presumably render these units more susceptible to an energy crisis both by increasing ATP consumption and by decreasing O_2_ and glucose supply, consequent to the elevated intramuscular pressure on the microvasculature supplying the contracting fibers. These small, overworked units were assumed to be more susceptible to an energy crisis and consequently to developing myofascial pain. They demonstrated the presence of such “underprivileged” units using electromyography recordings from human trapezius muscles.^30^

### Motor Unit Rotation

Recent studies cast some doubt on the ubiquitous adoption of the Cinderella hypothesis. In a series of elegant studies published during the last decade, De Luca and colleagues demonstrated a significant difference between the recruitment patterns of motor units in tonic muscles as compared to phasic ones.^29^,^31^,^32^ Tonic muscles are characterized by a predominance of slow-twitch fibers, which make them adapted to prolonged contractions and to maintaining the body posture in an anti-gravitational manner. Phasic muscles, which contain predominantly fast-twitch fibers, are more adapted to short contractions under high loads. De Luca shows that in phasic muscles (such as the distal muscles of the arm), which are adapted to intermittent contractions followed by periods of relaxation, motor units are indeed recruited in a *hierarchical pattern*: small first and large last, subscribing to the so-called “onion skin” pattern. However, in tonic, postural muscles (such as the deep muscles of the neck, back, and calves) motor units are recruited by *rotation* rather than by size, allowing them to contract and then to relax in a sequential shift-like manner. This pattern of recruitment seems advantageous since these muscles play a key role in postural stabilization and thus are required to contract for long periods of time, albeit with a sub-maximal load.

Motor unit rotation in postural muscles seems to pose a problem though: since recruitment in rotation precludes the presence of Cinderella units, the Cinderella hypothesis may not be a good model for myofascial pain in postural muscles, despite observations that these muscles are involved in myofascial pain more often than others.^3^ In this work, we suggest an alternative model for the development of myofascial pain in postural muscles, in which motor units are recruited in rotation.

### Hypothesis: The Shift Model

Both the energy crisis model and the Cinderella hypothesis implicitly assume that prolonged contraction of individual motor units may lead to the formation of myofascial trigger points, consequent to increased energetic demand and decreased oxygen supply during contraction. Here we hypothesize that motor unit *relaxation time* may be as important as *contraction time*, since it is during relaxation that motor units benefit from an improved microvascular blood flow and are thus able to replenish their oxygen and glucose supply and to dispose of noxious metabolites.^14^,^17^–^19^,^21^ We further hypothesize that a minimum relaxation/contraction time ratio is essential to allow the muscle fibers of a motor unit to recover. It seems reasonable to assume that any dysregulation of motor units’ recruitment pattern, leading to shorter relaxation periods and/or longer contraction periods, may result in an energy crisis and possibly the development of myofascial pain syndrome.

Interestingly, measurements performed in humans show altered unit rotation frequency in the trapezius muscles of healthy individuals exposed to postural and visual stressors.^33^

To test the plausibility of this hypothesis we created a model which aimed to explore the influence of known myofascial predisposing factors on motor unit relaxation/contraction time ratios.

The term the ‘shift model’ underlines our hypothesis that in postural muscles it is not necessarily the ‘Cinderella units’ which are prone to suffer from an energy crisis, but rather any unit, recruited in shifts, which does not get sufficient rest time.

## METHODS

We used a simple model to explore the correlation between muscle load, maximum muscle force, and the relaxation/contraction time ratio of motor units in a skeletal muscle. Let us consider a postural skeletal muscle during a prolonged isometric contraction. The muscle consists of *n* motor units. At any given time, the force generated by the muscle (equal to the load on the muscle) is dependent upon the number of motor units recruited and on their individual contraction force. Each motor unit is able to produce a different amount of force, depicted by *f_i_*. The fraction of recruited motor units (recruitment ratio) is depicted by *R*. The total force generated by the muscle, *F*, may be represented by Equation 1:

Equation 1 $$F=\sum_{i=1}^{R\cdot n}f_{i}$$

The motor unit firing rate modulation is not taken into account in this case, since a prolonged isometric contraction of a postural muscle is presumed.

The relaxation/contraction time ratio of an individual motor unit is presented in Equation 2, where *t_c_* is the average contraction time of a motor unit; *t_r_* represents the average relaxation time of a motor unit; and *R* is the recruitment ratio (ranging from 0 to 1):

Equation 2 $$\frac{t_{r}}{t_{c}}=\frac{1-R}{R}$$

### Numerical Modeling

Motor unit relaxation/contraction time ratios were modeled as follows: a model skeletal muscle was created, comprising 100 motor units, whose sizes were allotted in a power distribution with a predilection towards small units (see Dideriksen et al.^34^).

Recruitment of individual motor units during contraction was stochastic with a probability distribution that was either uniform or favored small units. Recruitment ratio was calculated recursively given the muscle load, using Equation 1. The average motor unit relaxation/contraction time ratios were then calculated using Equation 2.

The model was tested given a variety of numerical parameter values to ensure that the observed trends were independent of parameter values.

## MODEL RESULTS

### The Shift Model

As stated above, we hypothesize that motor unit relaxation time may play an important role in the development of an energy crisis. We further hypothesize that any dysregulation of motor units’ recruitment pattern, leading to shorter relaxation periods and/or longer contraction periods, may result in an energy crisis and possibly the development of myofascial pain syndrome.

To test these hypotheses, we developed the shift model, which simulates a postural skeletal muscle in which motor units are recruited in rotation. The muscle is isometrically contracted for prolonged periods of time. At any given time, the force generated by the contracted muscle is dependent upon the number of recruited motor units and their individual contraction forces (Equation 1). The relaxation/contraction time ratio of the individual motor units is only dependent upon the recruitment ratio. In fact, examining Equation 2 reveals an inverse correlation between the relaxation/contraction time ratio and the recruitment ratio, intuitively suggesting that the more units recruited, the shorter the relative relaxation time.

Looking further into Equation 1 reveals a positive correlation between the total muscle force and the recruitment ratio, and a negative correlation between individual motor unit force and the recruitment ratio. Intuitively, the higher the load on the muscle and the weaker the muscle, the more units are to be recruited.

Put together, the two equations imply a direct correlation between muscle strength and the relaxation/contraction time ratio; and an inverse correlation between muscle load and the relaxation/contraction ratio. In other words, it appears that insults, such as elevated muscle load and weakening of a muscle, lead to shorter relative relaxation times for each motor unit.

### Numerical Modeling

**Figure 1:**
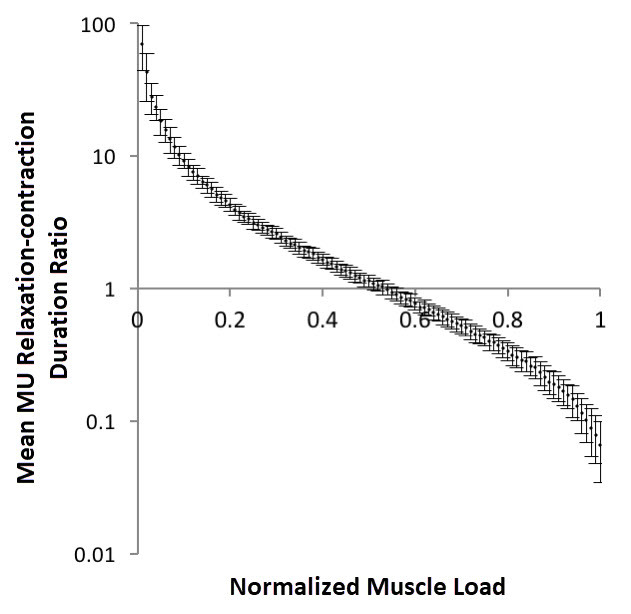
The Effect of Muscle Load on Average Motor Unit Relative Relaxation Time The figure represents the results of multiple simulated loadings of a single phasic muscle, with increasing loads, whose sizes are represented here as a fraction of the muscle’s maximum loading capacity (*x*-axis). Relative MU relaxation time, calculated as mean relaxation time/mean contraction time, is represented in the *y*-axis. Mean of 100 simulations, bars represent standard deviation. (MU, motor units).

To examine whether such insults could lead to a shortened relaxation/contraction time ratio, we created a simple numerical model. The model depicts a skeletal muscle in which motor units are recruited stochastically, in rotation (see Methods section for further details). First, we explored the effect of muscle loading on the relative relaxation time of motor units. Figure 1 shows a simulation in which the model muscle contracts under increasing loads, for which the subsequent motor unit relaxation/contraction time ratios are calculated. As intuitively expected, elevated loads on the model muscle lead to shorter motor unit relative relaxation times.

In fact, in this model, a load of ~50% of a muscle’s maximum contraction force would lead to a relaxation/contraction ratio of 1, meaning the average unit relaxes 1 second for each second it contracts.

**Figure 2:**
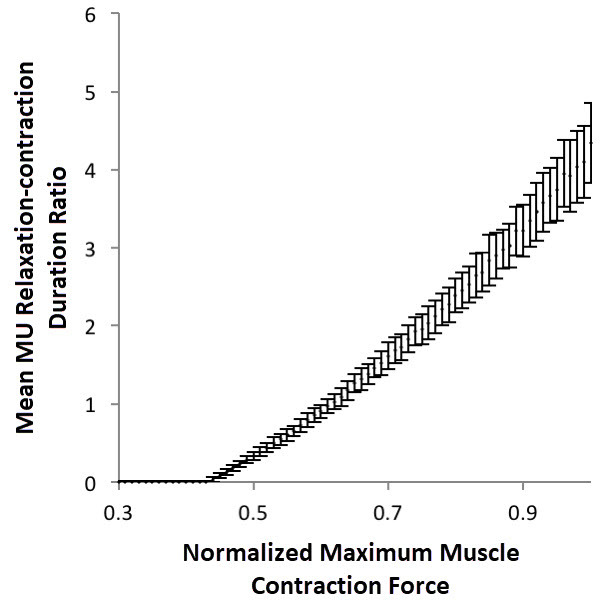
The Effect of Maximum Muscle Strength on the Average Motor Unit Relative Relaxation Time In this simulation, muscles of different maximum strength were loaded with a constant load, weighing 20% of the maximum muscle strength of the muscle presented in Figure 1. A mean MU relaxation/contraction duration ratio of 0 means that units are constantly contracted and the relaxation time equals 0. Mean of 100 simulations, bars represent standard deviation. (MU, motor unit).

Figure 2 shows a simulation in which muscles of different maximum strengths contract under a fixed load. Again, as expected, the weaker the muscle, the shorter the relative motor unit relaxation time.

**Figure 3:**
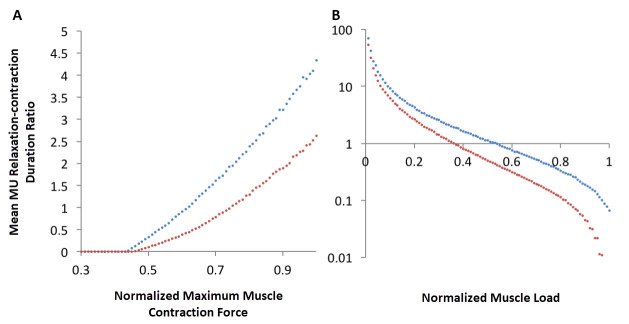
The Effect of Muscle Load on Average Motor Unit Relaxation Time (A) and the Effect of Muscle Strength on Average Motor Unit Relaxation Time (B) Uniform probability of recruitment distribution (blue); and small motor unit biased recruitment probability distribution (red). Note that the two curves are qualitatively similar. Mean of 100 simulations, error bars are not shown for the sake of clarity. (MU, motor unit).

These findings were not qualitatively dependent on different motor unit recruitment probability distributions that were simulated (Figure 3).

It is worth mentioning that this simplistic model is not expected to accurately represent motor unit recruitment in actual muscles, but rather to provide an indication as to the feasibility, at least in principle, of the model.

## DISCUSSION

In the present work, we describe a model that aims to bridge the gap between the widely accepted energy crisis theory and recent reports on motor unit rotation in postural muscles. Assuming that adequate relaxation time is essential for the energetic replenishment of motor units, we explored the influence of different factors on the average relaxation time of a motor unit. The main implications of the model are detailed below.

### Loaded or Weak Muscles are Prone to an Energy Crisis

Increased muscle load and decreased muscle force, both of which are well-known predisposing factors for myofascial pain syndrome, lead, under the conditions of the model, to the shortening of motor unit relaxation time, thus possibly making these motor units more susceptible to an energy crisis. The suggested causal correlation between muscle force, muscle load, and the development of an energy crisis might explain previous observations linking these factors to the development of myofascial pain. Thus, our model suggests a plausible physiological basis for the observation that weak or overloaded muscles are involved in myofascial pain syndrome more often than other muscles.

### The Energy Crisis as a Threshold Phenomenon

**Figure 4:**
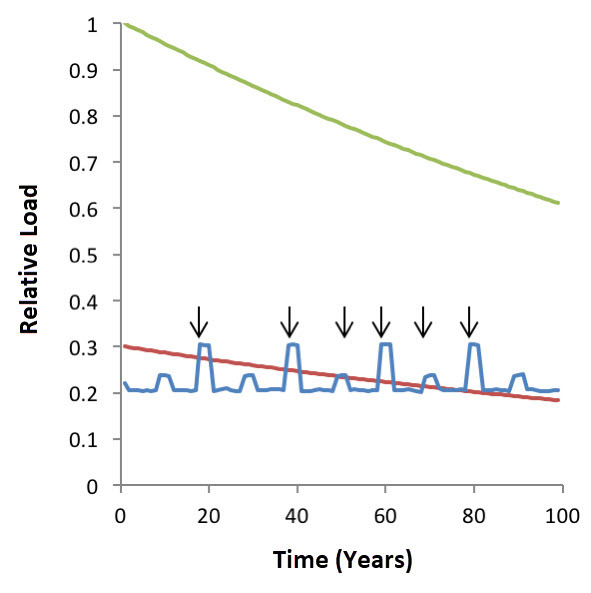
An Illustration of a Hypothetical Gradual Transition of Myofascial Pain to Chronicity Over Time Total muscle strength (green); threshold muscle load beyond which motor unit relaxation time falls below a critical value and an energy crisis occurs (red); actual load on the muscle (blue). Myofascial pain episodes occur when the muscle load exceeds the threshold load (arrows). These episodes increase in frequency as a muscle weakens, and eventually even low, everyday loads exceed the declining threshold, and chronicity ensues.

The proposed model implies that the energy crisis may be a threshold phenomenon that occurs once the ratio of relaxation/contraction durations falls short of a certain value. Beyond having significant implications for our understanding of myofascial pain etiology, this notion may also explain the syndrome’s occasional chronicity. A gradual path to chronic myofascial pain is observed in some patients: a first episode is followed by subsequent episodes at increasing frequency, sometimes leading to chronic pain. The representation of this process in our model may be of a healthy muscle, occasionally overloaded to a critical point in which motor unit relaxation times fall short of a threshold value, resulting in an energy crisis and myofascial pain. With time the muscle becomes weaker due to aging, immobility, or disease. The threshold for an energy crisis is lowered, and attacks become more frequent until, at a certain point, regular everyday loading exceeds the threshold, and chronicity ensues (Figure 4).

Of note, this model does not assume that dysregulation of motor unit recruitment is required for an energy crisis to develop. Rather, under certain stressors, such as muscle weakness or overload, the physiologic recruitment pattern, demonstrated in human and animal postural muscles, is sufficient to induce prolonged contraction and shortened relaxation times of individual motor units.

## STUDY LIMITATIONS

Being a purely theoretical model, this work warrants further *in vivo* experimental corroboration. The numerical parameter values used in this model have been adopted from previous theoretical studies, since few physiologically relevant *in vivo* measurements have been published. The significance of these results may therefore be regarded as qualitative rather than quantitative.

## CONCLUSIONS

Integrating the energy crisis hypothesis and recent observations on motor unit rotation, our model suggests a possible mechanism for the development of an energy crisis in postural skeletal muscles. The model suggests that in postural muscles, in which motor units are recruited in rotation, common predisposing factors for myofascial pain such as increased muscle load and decreased muscle force could lead to shortened motor unit relaxation periods and, subsequently, to an energy crisis, thus potentially resulting in the development of myofascial pain. The shift model described herein offers a possible causal relationship between muscle load, muscle strength, and the evolution of an energy crisis, as well as providing a logical mechanism for the threshold properties of the energy crisis phenomenon and, consequently, of the myofascial pain syndrome.
